# Effects of N and P addition on nutrient and stoichiometry of rhizosphere and non-rhizosphere soils of alfalfa in alkaline soil

**DOI:** 10.1038/s41598-023-39030-8

**Published:** 2023-07-26

**Authors:** Xudong Gu, Fengju Zhang, Xiaowei Xie, Yunlong Cheng, Xing Xu

**Affiliations:** 1 College of Agriculture, Ningxia University, Yinchuan, 750021 Ningxia China; 2 College of Ecology and Environment, Ningxia University, Yinchuan, 750021 Ningxia China

**Keywords:** Biogeochemistry, Chemical biology, Chemistry

## Abstract

Nitrogen (N) and phosphorus (P) are important nutrients for plant growth and development. Soil alkalization is one of the main obstacles limiting the sustainable development of agriculture. Northern Ningxia is located in the arid and semi-arid region, with serious soil alkalinization. Alfalfa has the advantages of strong saline-alkali tolerance, high yield, high quality, and wide adaptability. It is an important forage for the comprehensive improvement and rational utilization of saline-alkali land and has great significance for solving land resource shortages, improving the ecological environment, and ensuring food security. It is important to study soil organic carbon (SOC), total N (TN), total P (TP), and stoichiometry of the rhizosphere and non-rhizosphere of alfalfa in alkaline soils. Therefore, N and P were added to the alkaline alfalfa field in the Yinchuan Plain of Hetao Basin in our experiment. Six treatments were set up, i.e., N-free (WN), medium N (MN) for 90 kg/hm^2^, high N (HN) for 180 kg/hm^2^, P-free (WP), medium P (MP) for 135 kg/hm^2^, and high P (HP) for 270 kg/hm^2^. The results are as follows: The N addition promotes SOC and TN but inhibits TP, and P addition promotes SOC and TP but inhibits TN of three soil layers. The N addition decreases C/N but increases C/P and N/P, while the P addition increases C/N but decreases C/P and N/P of three soil layers. The SOC, TN, TP, C/N, C/P, and N/P under HN and HP treatment reach the significance level (*P* < 0.05). Appropriate additions of N and P can improve rhizosphere and non-rhizosphere nutrients and stoichiometric structure, facilitating absorption and utilization by alfalfa and improve the production potential of alfalfa in alkaline soil.

## Introduction

Salinization causes many problems, such as shortage of land resources, deterioration of the ecological environment, and decline of soil fertility^1^, which restricts the efficient use of the regional ecological environment and agriculture^2^. The saline-alkali land problem is a worldwide challenge^3^. Saline-alkali land is vast in China, covering an area of around 99.13 million hm^2^, or about 10% of the country’s land area^4^. Salinization is widespread across the country, especially in the arid and semi-arid region of the Hetao Plain, northwestern China, where agriculture and fragile ecological zones dominate. Due to the arid climate, intense evaporation, high salinity, and unreasonable irrigation system^5^, saline-alkali land area and salinization degree increase every year, and land degradation is serious, hindering the utilization of the land resources in Hetao Plain. Alfalfa is a perennial leguminous herbage with high yield, high protein, salt tolerance, cold resistance, and drought resistance^6–8^. Its cultivation can increase the content of nitrogen (N), phosphorus (P), and soil organic carbon (SOC), reduce the pH of saline-alkali soil^9^, and improve the ecological environment. Therefore, alfalfa cultivation is significant for biological improvement, land utilization, and environmental protection of saline-alkali land.

Soil carbon (C), N, and P are essential nutrients for plant growth, development, and reproduction^10^. They couple with each other, change dynamically in the cycle of soil nutrients, and maintain the nutrient balance of soil with microorganisms, enzymes, and metabolites^11,12^. As a structural substance, C is generally stable. The content of N and P is easily affected by external factors such as functional substances^13^, including climate, environment, land use, crop variety, and production management. The N and P content in the soil directly reflects soil fertility. Among them, N is highly mobile, especially nitrate N, which is absorbed and utilized by the root system in ionic form. Through experiments on the effect of N addition on soil C content, Wang et al. and Cusack et al. found that N addition increased soil C content^14,15^. Su et al.^16^ found that N addition can increase soil C content and decrease soil pH. In addition, P has poor mobility and can be classified into water-soluble P, acid-solubility P, and insoluble P. The application of P can increase available P content and effectively allocate the nutrient cycle of P in soils^17^. Wekha et al.^18^ found that P addition decreased soil pH and increased TN of soil. Peng et al. also found that P addition reduced the pH of alfalfa fields in alkaline soil^19^. Therefore, N and P addition can significantly improve the quality of alkaline soil. Moreover, C and P are components of many important compounds, such as chlorophyll, nucleic acid, protein, and enzyme, which are involved in metabolic processes such as photosynthesis, respiration, storage and transmission of energy, and cell division. N and P are important for the growth and development of alfalfa.

Ecological stoichiometry examines the balance relation of chemical elements (mainly N, P, and C) during ecological interaction^20,21^ and is a powerful tool for studying the coupling, stoichiometric homeostasis, and biogeochemical cycling of C, N, and P in the ecosystem^22,23^. C/N, C/P, and N/P reflect the fertility and stability of the soil. Scholars have researched different ecosystems, such as sandy land, wetland, and forest land. Ecological stoichiometry has been developed into a new tool for biology research at different scales, including molecular biology, cell, population, and ecosystem, and it varies depending on different ecosystems^24,25^. Ma et al.^26^ found that P addition can increase the N/P ratio in low-altitude rain-forest soil of Xishuangbanna and decrease the N/P ratio in high-altitude rain-forest soil of Xishuangbanna, thus limiting N or P in Xishuangbanna rain-forest soil. Song et al.^27^ found that the C/N and N/P of farmland on the Loess Plateau were significantly influenced by N and P and varied with their annual increase. Ecological stoichiometry is significant in studying nutrient limitation and balance of soil^28^.

At present, there are few single-factor experiments on N and P of alfalfa in alkaline land, especially experiments on the effects of N and P addition on the stoichiometry of rhizosphere and non-rhizosphere soils of alfalfa in the alkaline field. In this study, N and P experiments were conducted in the alkalized alfalfa field of northern Ningxia to study the changing characteristics of nutrient content and stoichiometry in the rhizosphere and non-rhizosphere soils in Yinchuan Plain of Hetao Basin with the comprehensive factors of fertilization, alkaline soil, and alfalfa. This study aimed to provide theoretical guidance for scientific fertilization of alfalfa in the alkaline field and prevent the limitation and waste of alfalfa growth nutrients caused by unreasonable fertilization, thus improving the economic effect, living environment, and utilization of land resources.

## Materials and methods

### Site description

**Figure 1:**
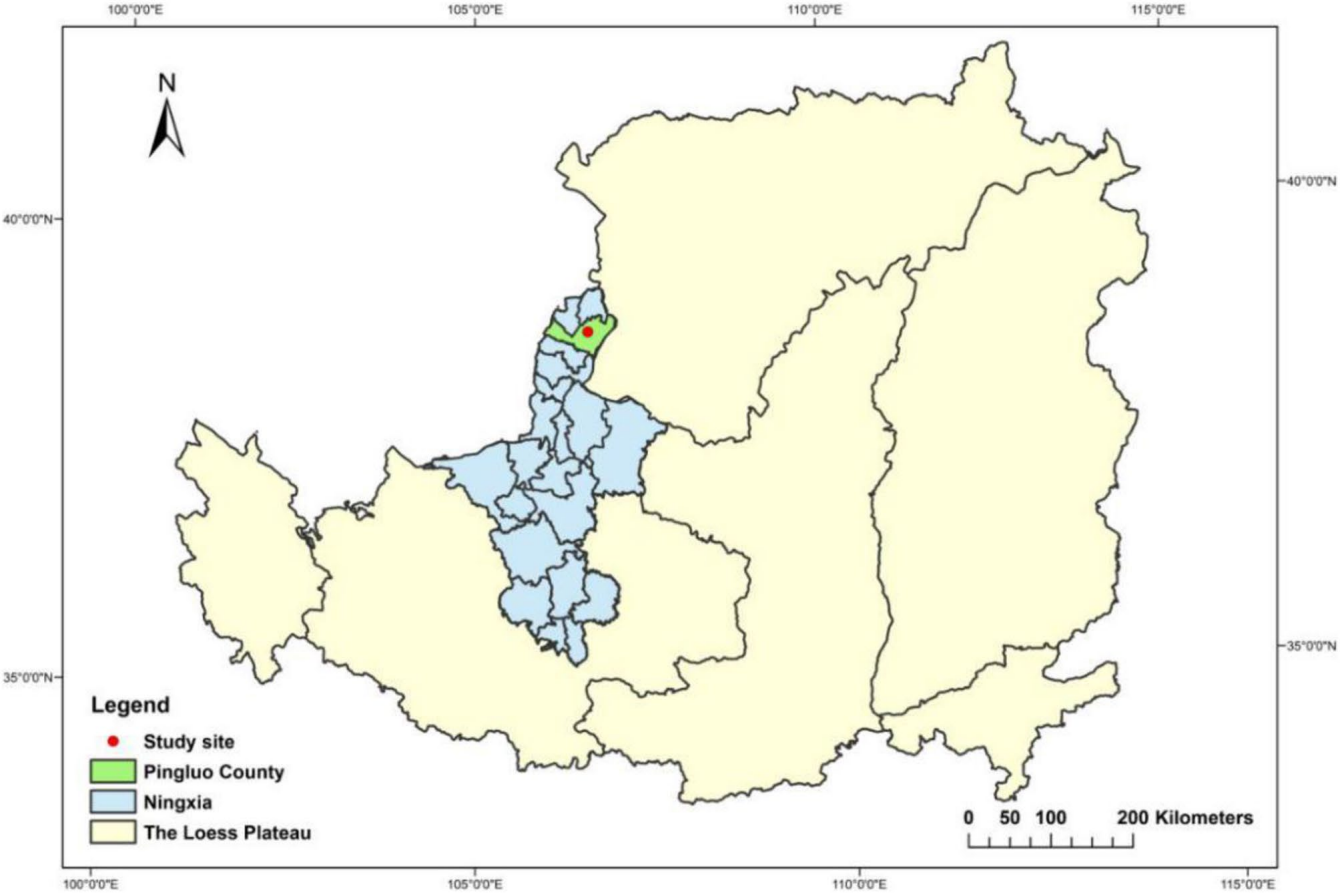
Location map and research site of study (ARCGIS10.2 used for drawing).

The experiment was performed at the test base of Ningxia Qianye Qing Agricultural Technology Development Co., Ltd (Pingluo County, Ningxia, China) in early July 2019 (38° 50’ N, 106° 15’ E; 1091–1110 m above sea level). The research site and map of this study are shown in Fig. 1. Pingluo is located in a drought and desert area in China. The solar radiation is 4225.9 kJ/m^2^ from April to October. The annual cumulative sunshine is between 2800 and 3200 h, with the longest sunshine hours in June. Pingluo has little rainfall and high evaporation. Its annual precipitation is 200–270 mm, with 66.6% of the precipitation from July to September. The most amount of evaporation range from 1500 to 1800 mm. The average annual humidity is about 56%. The average annual temperature is about 6.8 °C, and the annual effective accumulated temperature of 10 °C and above is 1638 °C–2638 °C. Additionally, the frost-free period is about 150 days. The main soil in the tested field is alkaline soil, and salt ions are mainly Na^+^, Cl^−^, and SO_4_^2−^. The physical and chemical properties of the tested soil at different depths are shown in Table 1.

**Table 1:** Physical and chemical characteristics of the tested soil.

Depth	Index									
	SOC (g/kg)	OM (g/kg)	TN (g/kg)	NH_4_^+^_-_N (mg/kg)	NO_3_^−^_-_N (mg/kg)	TP (g/kg)	AP (mg/kg)	AK (mg/kg)	ESP (%)	pH
20 cm	16.04	27.65	1.18	11.30	193.96	5.34	112.91	126.97	12.32	8.40
40 cm	10.52	18.14	0.97	7.41	256.21	3.92	42.06	130.99	14.47	8.50
60 cm	8.12	14.00	0.86	9.12	286.87	3.14	121.57	143.05	16.87	8.57

The SOC, OM, TN, NH_4_^+^_-_N, NO_3_^−^_-_N, TP, AP, TK, ESP, AK refer to organic carbon, organic matter, total nitrogen, ammonium nitrogen, nitrate nitrogen, total phosphorus, available phosphorus, alkalinity, available potassium respectively.

### Experiment design and field management

**Figure 2:**
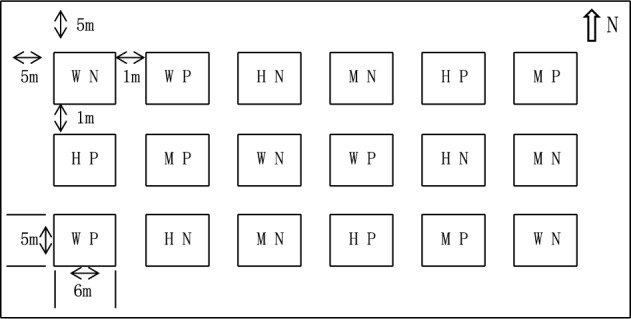
Field layout of nitrogen and phosphorus treatment.

The experiment was conducted in a randomized block design with six treatments, including N-free (WN), medium N (MN), high N (HN), P-free (WP), medium P (MP), and high P (HP). The application rates of WN, MN, and HN were 0, 90, and 180 kg/hm^2^, with 120 and 75 kg/hm^2^ of P and potassium rates as supporting fertilizers for N treatments. The application rates of WP, MP, and HP were 0, 135, and 270 kg/hm^2^, with 90 and 75 kg/hm^2^ of N and potassium rates as supporting fertilizers for P treatments. Experiments were replicated 3 times with 18 plots. Artificial ridging technology was applied for plots. Plots were spaced 1 m apart, and the guard row of the test field was 5 m. The area of a single plot was 30 m^2^ (5 m × 6 m), the area of the experiment was 540 m^2^, and the actual area was 970 m^2^ (Fig. 2). In mid-April 2017, 22.5 kg/hm^2^ of alfalfa was mechanically sown, with a row spacing of 30 cm. Fertilizers were manually added. N was applied twice a year in April and July, while P was applied once a year in April. Supporting fertilizers were applied once a year in April. The alfalfa variety is the Salt-tolerant Star (from Ningxia Qianye Qing Agricultural Technology Development Co., Ltd.). The N fertilizer is urea (N ≥ 46.4%), The P fertilizer is heavy calcium superphosphate (P_2_O_5_ ≥ 46%), and the potash fertilizer is potassium sulfate for agricultural use (K_2_O ≥ 50%). Alfalfa was harvested artificially four times on June 10, July 15, August 20, and October 10, leaving 5 cm of stubble. Watering was performed four times on May 15, June 15, July 15, and August 15 at a depth of 5 cm. In addition, other relevant management measures are consistent.

### Sampling and analysis

#### Sampling

Rhizosphere and non-rhizosphere soil samples of alfalfa were collected at the early flowering of the second alfalfa crop in June each year. Non-rhizosphere soils of alfalfa were collected using the method described by Bao^29^. According to the five-point method (the midpoint of the diagonal of the test plot was used as the central sampling point, and then four points on the diagonal with equal distance from the central sampling point were selected as sampling points), the non-rhizosphere soil samples were collected using a soil auger (approximately 5 cm in diameter). The collection of the non-rhizosphere soil samples was performed at 20 cm intervals in the 0–20 cm, 20–40 cm, and 40–60 cm soil layers up to a depth of 60 cm from the surface. The rhizosphere soils of alfalfa at 20 cm were collected according to Gqozo et al.^30^, and 10–15 well-grown alfalfa plants were selected from each collection point. After uprooting the alfalfa, the soil attached to its roots (rhizosphere soil) was collected by shaking the root. The collected soil samples were air-dried, ground, and filtered through 0.25, 1, and 2 mm sieves for laboratory analysis.

#### Analysis

According to the soil texture classes introduced by The United States Department of Agriculture, the soil was divided into sand (0.05–2 mm), silt (0.002–0.05 mm), and clay (< 0.002 mm). In this study, soils passing through the 1 mm mesh sieve were used to determine ammonia N (NH_4_^+^_-_N), nitrate N (NO_3_^−^_-_N), available P (AP), and available potassium (AK); those passing through the 0.25 mm mesh screen were used to measure organic C (OC), total N (TN), total P (TP), and exchangeable sodium percentage (ESP).

The contents of soil OC, TN, and AP were analyzed by the potassium dichromate-external heating method, semi-microkelvin method, and molten NaOH-Molybdenum-antimony resistance colorimetric method^31^. Soil OM content was obtained by multiplying OC content by a conversion factor of 1.724^29^. Quantification of soil AK content was performed by flame photometry with 1 mol/L ammonium acetate extract (PE PinAAciie 900F, America). Soil TP content was analyzed by molybdenum antimony blue calorimetry^32^. After extraction with 2 mol/L KCl, a continuous flow analyzer (AutoAnalyzer-AA3, Seal Analytical, Norderstedt, Germany) was used to measure the soil NH_4_^+^_-_N and NO_3_^−^_-_N^33^. The soil pH was measured using a pH meter (Mettler-Toledo FE 20; Switzerland) at a soil/water ratio of 1:2.5 after approximately 30 min of shaking the equilibration^34^. Exchangeable Na^+^ was extracted using ammonium acetate, and its concentration was measured by Prodigy-7 ICP-AES. Based on the equation (*ESP* = (*Na*^+^*/CEC*) × 100) proposed by Seilsepour et al.^35^, the ESP in this study was calculated after analyzing the cation exchange capacity (CEC) using the BaCl_2_ and NH_4_OAc methods.

##### Analysis of SOC (volumetric method of potassium dichromate-external heating method)

Principle of analysis: Under external heating conditions (oil-bath temperature of 180 °C, boiling for 5 min), the SOC was oxidized with the solution of potassium dichromate and sulfuric acid solution at a certain concentration, and the remaining potassium dichromate was titrated with ferrous sulfate. The content of SOC was calculated based on the consumption of potassium dichromate.

$${\text{Calculation}}\;{\text{formula}}:{\text{ SOC}}\left( {{\text{g}}\cdot{\text{kg}}^{{ - {1}}} } \right) = \left[ {\left( {{\text{c}} \times {5}} \right) \, /{\text{Vo}} \times ({\text{Vo}} - {\text{V}}) \times { 1}0^{{ - {3}}} \times {3}.0 \times {1}.{1}} \right] \times {1}000/\left( {{\text{m}} \times {\text{k}}} \right)$$

where c, 0.8000 mol L^−1^ (1/6 K_2_Cr_2_0_7_) of standard solution; 5, Volume of standard solution with potassium dichromate (mL); Vo, FeSO_4_ volume used by blank titration (mL); V, FeSO_4_ volume used by sample titration (mL); 3.0, 1/4 molar mass of carbon atom (g mol^−1^); 10^−3^, Conversion of mL to L; 1.1, Correction coefficient of oxidation; m, Quality of air-dried soil sample (g); k, Coefficient of conversion of air-dried soil into baked soil.

##### Analysis of TN (semi-microkelvin method)

Principle of analysis: samples were dissolved and boiled with concentrated sulfuric acid by accelerators, and various N-containing organic compounds were converted into ammonium through complex pyrolysis reactions. The ammonium sulfate was obtained after the combination of ammonium with sulfuric acid. Finally, the converted ammonia was absorbed with boric acid and titrated with a standard acid solution to analyze the TN content of samples.

$${\text{Calculation formula}}:{\text{ TN}}\left( {{\text{g}}\cdot{\text{kg}}^{{ - {1}}} } \right) = \left[ {\left( {{\text{V}} - {\text{Vo}}} \right) \times {\text{c}}\left( {{1}/{\text{2H}}_{{2}} {\text{SO}}_{{4}} } \right) \times {14}.0 \times {1}0^{{ - {3}}} } \right] \times {1}0^{{3}} /{\text{m}}$$

where V, Volume of acid standard solution used in the tested solution of titration (mL); Vo, Standard volume of acid used in the titration of blank (ml); c, 0.01 mol L^−1^ (1/2H_2_SO_4_) or HCI standard solution concentration; 14.0, Molar mass of N atom (g mol^−1^); 10^−3^, Conversion of mL to L; m, Quality of baked soil samples (g).

##### Analysis of TP (molten NaOH-Molybdenum-antimony resistance colorimetric method)

Principle of analysis: After melting the soil sample with sodium hydroxide, the phosphorous minerals and organic P compounds were converted into soluble orthophosphates, and the frits were dissolved with water and dilute sulfuric acid. The P molybdenum blue was generated by reacting the sample solution with the reagent of molybdenum-antimony color, followed by the quantitive analysis through spectrophotometry.

$${\text{Calculation formula}}:{\text{ TP}}\left( {{\text{g}}\cdot{\text{kg}}^{{ - {1}}} } \right) = \rho \times ({\text{V}}_{{1}} /{\text{m}}) \times ({\text{V}}_{{2}} /{\text{V}}_{{3}} ) \times {1}0^{{ - {3}}} \times [{1}00/({1}00 - {\text{H}})]$$

where ρ, Mass concentration of P in the sample solution to be tested that can be obtained from the calibration curve (mg L^−1^); m, Sample weight (g); V_1_, Constant volume of the sample after melting (ml); V_2_, Constant volume of solution during color development (mL); V_3_, Volume extracted from the molten sample after constant volume (mL); 10^−3^, Conversion factor of concentration unit of mg L^−1^ to mass unit of kg; 100/(100 − H), Conversion factor of air-dried soil to baked soil; H, Percentage of moisture content in air-dried soil.

The C/N, C/P, N/P, C, N, and P refer to organic C/TN, organic C/TP, TN/TP, organic C, TN, and TP, respectively.

### Data processing and statistical analysis

All treatments were independently (or biologically) performed in triplicate. Data were sorted by Microsoft Excel 2019 software and processed by SPSS 19.0 and Origin 2019 software. Among them, SPSS 19.0 was used to analyze the significant differences in nutrients and stoichiometry of rhizosphere and non-rhizosphere soils. Analysis of variance (ANOVA) was applied for hypothesis testing. *P* < 0.05 was considered statistically significant. ArcGIS10.2 software was used to plot Fig. 1.

## Results

### Effects of N and P addition on the nutrient accumulation in the rhizosphere and non-rhizosphere soils of alfalfa

**Figure 3:**
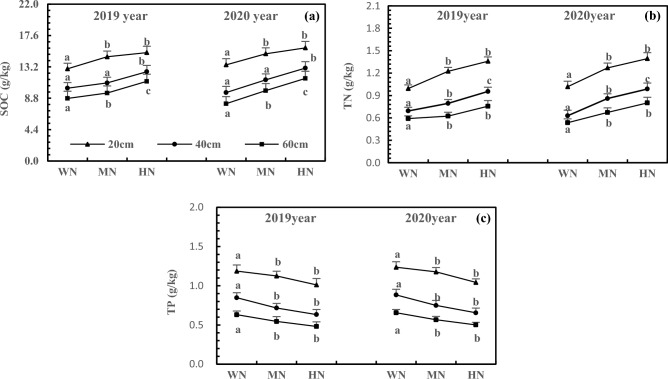
Effects of N addition on SOC (**a**), TN (**b**) and TP (**c**) accumulation of three-layer soils. Note: 1. The SOC, TN and TP refer to soil organic carbon, total nitrogen and phosphorus of three-layer soils of non-rhizosphere, respectively. 2. Different lowercase letters above the lines refer to significant differences among different treatments of same soil layer at *P* < 0.05 level. 3. The note below are the same.

According to supplementary information 1, the accumulation of SOC, TN, and TP in the three-layer soils in 2020 was similar to that in 2019 after the addition of N. Compared to the 40 and 60 cm soils, the 20 cm soil had a slightly higher accumulation of SOC, TN, and TP, while the differences between 40 and 60 cm soils were relatively small (Fig. 3). Moreover, the SOC, TN, and TP accumulation after HN treatment were significant compared with that after WN treatment in the three-layer soils (*P* < 0.05). With increasing N addition, SOC and TN accumulation increased, while TP accumulation decreased at each soil depth. In 2020, the HN treatment led to 19.37%, 33.72%, and 62.96% higher SOC accumulation in soils at the three depths compared to the WN treatment (Fig. 3a). The TN accumulation after the HN treatment increased by 36.99%, 56.89%, and 49.42% in soils at the three depths (Fig. 3b). In contrast, the TP accumulation showed a reduction of 15.67%, 25.82%, and 23.58% after HN treatment the three depths of the soil (Fig. 3c).

**Figure 4:**
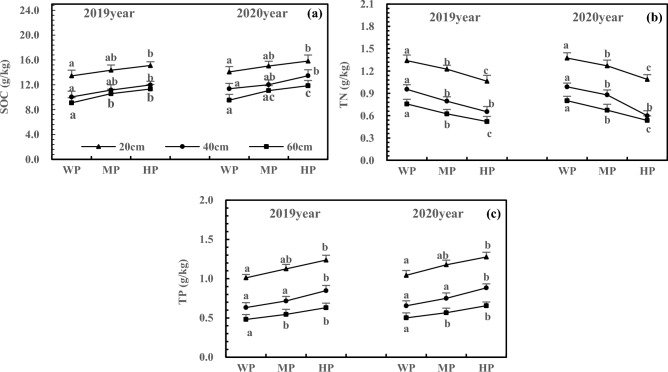
Effects of P addition on SOC (**a**), TN (**b**) and TP (**c**) accumulation of three-layer soils.

The P addition resulted in similar SOC, TN, and TP accumulation in the three-layer soils after HP treatment in 2020 as in 2019. The accumulation of SOC, TN, and TP was slightly higher in 20 cm soil than in 40 and 60 cm soils, while the difference between 40 and 60 cm soils was relatively small. Compared with the WP treatment, the HP treatment led to significant differences (*P* < 0.05) in the accumulation of SOC, TN, and TP in the three-layer soils. With increasing P addition, the accumulation of SOC and TP and that of TN decreased in soils at all depths (Fig. 4). In 2020, the SOC accumulation increased by 12.43%, 18.36%, and 24.46% in soils at the three depths after HP treatment than after WP treatment (Fig. 4a). The TN accumulation after HP treatment decreased by 20.84%, 29.17%, and 33.07% in soils at the three depths (Fig. 4b). Furthermore, the TP accumulation increased by 22.41%, 34.81%, and 30.85% in soils at the three depths after HP treatment (Fig. 4c).

**Figure 5:**
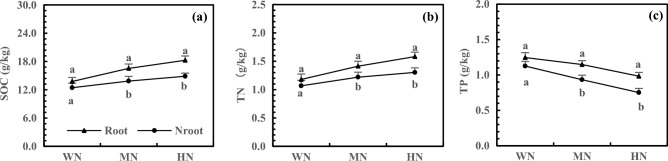
Effects of N addition on SOC C (**a**), TN (**b**) and TP (**c**) accumulation of rhizosphere and non-rhizosphere soils. Note: 1. The SOC, TN and TP refer to soil organic carbon, total nitrogen and phosphorus of 20 cm depth of rhizosphere and non-rhizosphere soils respectively. 2. Different lowercase letters above same treatment represent significant differences between rhizosphere and non-rhizosphere soils at *P* < 0.05 level. 3. Root and Nroot stand for rhizosphere and non-rhizosphere respectively. 4. The note below are the same.

As the N addition increased, SOC and TN accumulation increased in both rhizosphere and non-rhizosphere soils, but TP accumulation showed an increasing trend. The accumulation of SOC, TN, and TP in rhizosphere soils reached the significance level under MN and HN treatments (Fig. 5, *P* < 0.05). Compared with WN treatment, HN treatment resulted in 32.66% and 19.54% higher SOC accumulation in the rhizosphere and non-rhizosphere soil, respectively. The accumulation of SOC is 22.80% higher in the rhizosphere soils than in non-rhizosphere soils under HN treatment (Fig. 5a). As shown in Fig. 5b, the TN accumulation in the rhizosphere and non-rhizosphere soils increased by 34.09% and 22.15% after the NH treatment, respectively, with 21.32% higher TN accumulation in rhizosphere soils than in non-rhizosphere soils. Furthermore, NH treatment increased the TP accumulation by 21.10% and 33.19% in rhizosphere and non-rhizosphere soils, respectively, with 30.67% higher TP accumulation in rhizosphere soils than in non-rhizosphere soils (Fig. 5c).

**Figure 6:**
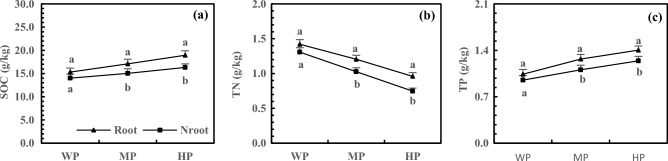
Effects of P addition on SOC (**a**), TN (**b**) and TP (**c**) accumulation of rhizosphere and non-rhizosphere soils.

The accumulation of SOC and TP increased in the rhizosphere and non-rhizosphere soils with increasing P addition, but the TN accumulation showed the opposite trend. Under MP and HP treatments, the SOC, TN, and TP accumulation in rhizosphere soils reached the significance level (Fig. 6, *P* < 0.05). The HP treatment increased the SOC accumulation in the rhizosphere and non-rhizosphere soils by 23.66% and 16.48%, respectively, with 16.08% higher SOC accumulation in rhizosphere soils than in non-rhizosphere soils (Fig. 6a). Compared with WP treatment, the HP treatment resulted in 32.49% and 42.85% lower TN accumulation in the rhizosphere and non-rhizosphere soils, respectively, and with 28.35% higher accumulation of TN in rhizosphere soils than in non-rhizosphere soils (Fig. 6b). Moreover, the TP accumulation increased by 21.10% and 33.19% after HP treatment in the rhizosphere and non-rhizosphere soils, respectively, with 30.67% higher TP accumulation in rhizosphere soils than in non-rhizosphere soils (Fig. 6c).

### Effects of N and P addition on nutrient stoichiometry in the rhizosphere and non-rhizosphere soils of alfalfa

**Figure 7:**
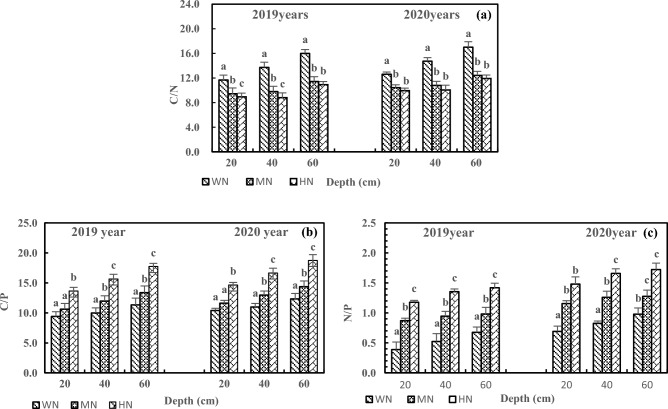
Effects of N addition on C/N (**a**), C/P (**b**) and N/P (**c**) of three-layer soils. Note: 1. The C/N, C/P and N/P refer to carbon–nitrogen ratio, carbon-phosphorus ratio and nitrogen-phosphorus ratio of three-layer soils of non-rhizosphere respectively. 2. Different lowercase letters above the bars on same layer represent significant differences among different treatments at *P* < 0.05 level. 3. The note below are the same.

According to supplementary information 2, after the addition of N, the C/N, C/P, and N/P in the three-layer soils in 2020 were similar to those in 2019. The changes became more significant with increasing soil depth. The N addition decreased the C/N and increased the C/P and N/P in the same layer. Under HN treatment, C/N, C/P, and N/P reached the significance level in the same layer (Fig. 7, *P* < 0.05). In 2020, the HN treatment decreased the C/N by 21.20%, 31.79%, and 29.94% in 20, 40, and 60 cm soils, respectively (Fig. 7a), increased C/P by 40.77%, 51.61%, and 51.93% in 20, 40, and 60 cm soils, respectively (Fig. 7b), and increased N/P by 113.97%, 100.58%, and 76.01% in 20, 40, and 60 cm soils, respectively (Fig. 7c).

**Figure 8:**
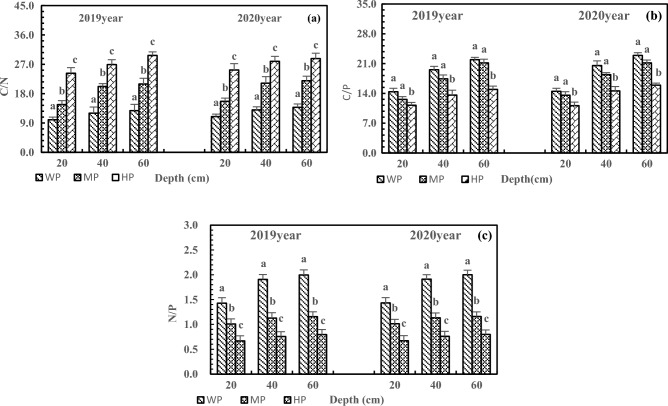
Effects of P addition on C/N (**a**), C/P (**b**) and N/P (**c**) of three-layer soils.

With the addition of P, the C/N and C/P in the three-layer soils in 2020 were similar to those in 2019, but N/P showed the opposite trend. Furthermore, P addition increased the C/N of the same layer but decreased the C/P and N/P. The C/N, C/P, and N/P of the same layer reached the significance level under HP treatment (Fig. 8, *P* < 0.05). In 2020, the C/N increased by 129.84%, 114.67%, and 108.16% in 20, 40, and 60 cm soils after HP treatment, respectively (Fig. 8a), C/P decreased by 15.49%, 29.00%, and 30.51% in 20, 40, and 60 cm soils, respectively (Fig. 8b), and N/P decreased by 53.11%, 60.19%, and 60.01% in 20, 40, and 60 cm soils, respectively (Fig. 8c).

**Figure 9:**
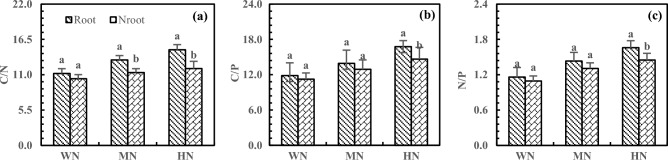
Effects of N addition on C/N (**a**), C/P (**b**) and N/P (**c**) of rhizosphere and non-rhizosphere soils. Note: 1. The C/N, C/P and N/P refer to carbon–nitrogen ratio, carbon-phosphorus ratio and nitrogen-phosphorus ratio of 20 cm depth of rhizosphere and non-rhizosphere soils respectively. 2. Different lowercase letters above the bars on same treatment represent significant differences between rhizosphere and non-rhizosphere soils at *P* < 0.05 level. 3. The note below are the same.

The N addition increased the C/N, C/P, and N/P in rhizosphere and non-rhizosphere soils. The C/N, C/P, and N/P were higher in rhizosphere soils than in non-rhizosphere soils, reaching the significance level under the HN treatment (Fig. 9, *P* < 0.05). Compared with WN treatment, HN treatment led to 32.93% and 15.26%higher C/N in the rhizosphere and non-rhizosphere soils, respectively, with 24.49% higher C/N in rhizosphere soils than in non-rhizosphere soils (Fig. 9a). The C/P increased by 41.57% and 30.36% in the rhizosphere and non-rhizosphere soils after HN treatment, respectively, with 14.40% higher C/P in rhizosphere soils than in non-rhizosphere soils (Fig. 9b). Furthermore, the HN treatment increased the N/P by 44.75% and 30.85% in the rhizosphere and non-rhizosphere soils, respectively, with 17.37% higher C/P in rhizosphere soils than in non-rhizosphere soils (Fig. 9c).

**Figure 10:**
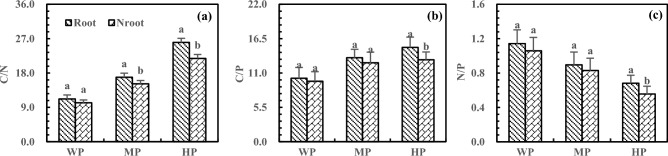
Effects of P addition on C/N (**a**), C/P (**b**) and N/P (**c**) of rhizosphere and non-rhizosphere soils.

The P addition increased C/N and C/P in the rhizosphere and non-rhizosphere soils but decreased N/P (Fig. 10). Under HP treatment, the C/N, C/P, and N/P were higher in rhizosphere soils than in non-rhizosphere soil and reached the significance level (*P* < 0.05). Compared with WP treatment, HP treatment led to 132.20% and 113.50% higher C/N in the rhizosphere and non-rhizosphere soils, respectively, with 19.26% higher C/N in rhizosphere soils than in non-rhizosphere soils (Fig. 10a). The C/P increased by 48.71% and 35.51% in the rhizosphere and non-rhizosphere soils after HP treatment, respectively, with 15.14% higher C/P in rhizosphere soils than in non-rhizosphere soils (Fig. 10b). Moreover, the HP treatment decreased N/P by 40.31% and 47.47% in the rhizosphere and non-rhizosphere soils, respectively, with 22.56% higher N/P in rhizosphere soils increased than in non-rhizosphere soils (Fig. 10c).

## Discussion

In this study, N and P addition increased SOC in three soils, reaching a significance level after HN and HP treatments (*P* < 0.05). Although the structural element C is relatively stable and generally unaffected by nutrient availability, it is significantly influenced by root exudation, which may be related to root exudates of alfalfa. Shen et al. studied the temperate grassland in Inner Mongolia and demonstrated that root exudation could promote C accumulation^36^. Additionally, N and P addition improved root growth and spatial distribution^37,38^ increased root exudates, resulting in an increased SOC. The increase in C accumulation is also related to the inhibition of SOC mineralization by salinization^39^. In this study, N addition increased TN in three soils and decreased TP, while the addition of P caused the opposite result. Furthermore, TN and TP reached the significance level under HN and HP treatments (*P* < 0.05). The accumulation of functional N and P was greatly affected by nutrient supply, which was the main reason for the limitation of P or N. Huang et al. revealed the effect of atmospheric N deposition in increasing soil TN and decreasing soil TP^40^, which is consistent with the result of this study. The rhizosphere is the core zone of plants, microorganisms, and soils, with frequent material circulation and energy flow^41^. In this study, SOC, TN, and TP were higher in rhizosphere soils than in non-rhizosphere soils and reached the significance level (*P* < 0.05) after HN and HP treatments. This result is consistent with the study on peanut and flue-cured tobacco^42^ and is related to the composition of various substances that affect the root system, such as exudation, enzyme, and microorganism^43,44^.

The C/N, C/P, and N/P reflect nutrient absorption, preservation, decomposition, and limitation^45,46^, and these ratios are affected by the addition of N and P. In this study, the addition of N and P increased C/N, C/P, and N/P in the deep soil, leading to N and P limitation or wastage, disrupting the nutrient balance, and affecting nutrient absorption and utilization in alfalfa. Due to the rhizosphere enrichment^47^, the addition of N increased the C/N in rhizosphere soils and decreased the C/N in non-rhizosphere soils. Tang et al. found that microbial biomass C was higher in rhizosphere soils than in non-rhizosphere soils^48^. Moreover, N addition greatly increased the C/N in the deep soil, which was different from the result of most studies and might be related to the characteristics of saline-alkali soil and alfalfa. He et al. reported that responses of root length and rhizosphere carboxylates depended on soil type^49^. Furthermore, P addition increased the C/N in the rhizosphere and non-rhizosphere soils and caused N limitation in rhizosphere soils. This result was mainly due to rhizosphere enrichment, P addition, and alkaline soil. The previous study by He et al. demonstrated that P addition could increase the loss of N in P-poor soil^50^, which supports the present study. The C/N value in Chinese soil is between 10 and 14^51^. Too high or too low additions of N and P can break the balance of C/N, which is detrimental to the release and absorption of soil nutrients by alfalfa. Liu et al. found that proper fertilization is beneficial to the utilization and distribution of soil nutrients by plants^52^. Therefore, proper N and P addition can promote the balance and coupling of N and P in soil and facilitate the absorption and utilization of N and P by alfalfa.

In this study, the addition of N and P under WN and HP treatments kept the C/P around 10 in the soil at 20 cm depth^51^. Excessive N or insufficient P may result in waste of C or limitation of P, which is detrimental to nutrient uptake and utilization by alfalfa. In addition, N and P increased C/P in the deep soil because N promoted the absorption of P. Amador et al. found that the effects of soil moisture on C metabolism depended on the availability of C/P and C, and C/P affected C mineralization^53^. By studying the effects of N addition on the stoichiometry of soil in Stipa baicalensis grassland, Liu et al.^55^ reported that N addition could increase C/P in soil^54^, which is consistent with the present study. Under HN and HP treatments, C/P reached the significance level (*P* < 0.05) in rhizosphere soils, which is inconsistent with the findings of Liu that low N and P addition could promote soil SOC in the meadow of Qinghai-Tibet Plateau. This inconsistency is associated with the combined effects of alfalfa, alkaline soil, and fertilizer. The pH is lower in rhizosphere soils than in non-rhizosphere soils, and rhizosphere soils are prone to acidification^56,57^. Ding et al.^58^ and Gillespie et al.^59^ found that acidified rhizosphere soil was more likely to promote mineralization and uptake of P by plants, which could explain the result of the present study. Increased C/P is not conducive to the balance of C and P and tends to cause C wastage and P deficiency. Therefore, MN and MP are more conducive to the balance of C and P in alkaline rhizosphere soils of alfalfa.

N/P is highly susceptible to environmental nutrient supply, especially the addition of N and P. The addition of N leads to an increase in N/P in the same and deep soils due to the increased N and the limitation of P. As a result, N wastage and P deficiency occur in the deep soil, which is detrimental to the N and P balance of soil and absorption of alfalfa. By studying the effect of N addition on soil stoichiometry in Mexican Cypress plantations, Li et al.^60^ found that N addition increased N/P. He et al.^61^ found that N addition led to a higher deficiency of P. Due to the addition and fixation of P in upper soil layers, P addition decreased N/P, and N/P increased with soil depth, which is consistent with the experiment performed by Ma et al. in Xishuangbanna^26^. Chen et al.^62^ reported that P availability is mainly a7ffected by pH, which easily leads to the limitation of P in deep soil. The N/P of rhizosphere soils reached a significance level (*P* < 0.05) under HN treatment, which may be related to the increased N content by the N fixation of alfalfa. Ye et al.^63^ also found that N addition improved the N fixation of alfalfa. The N/P is significantly higher in rhizosphere soils than in non-rhizosphere soils (*P* < 0.05) after HP treatment, indicating that P promotes N fixation in rhizosphere soils. Crews conducted experiments on the effect of P addition on the N fixation of alfalfa in Mexico. The results showed that P addition promoted the N fixation of alfalfa^64^. The increased N/P can also cause an imbalance of N and P in the rhizosphere soils. Therefore, excessive N or P addition can disrupt the nutrient balance and cause limitation of P or N in the rhizosphere^65,66^, affecting the absorption and utilization by alfalfa.

Based on the above results, excessive N and P addition can harm alfalfa growth, resulting in fertilizer waste and environmental pollution in alkaline soil. Appropriate additions of N and P can improve rhizosphere and non-rhizosphere nutrients and stoichiometric structure, thus facilitating absorption and utilization by alfalfa and improving the production potential of alfalfa in alkaline soil.

## Conclusion

The N addition promoted SOC and TN but inhibited TP, and the P addition promoted SOC and TP but inhibited TN in the three soil layers. Moreover, the N addition decreased C/N but increased C/P and N/P, while the P addition increased C/N but decreased C/P and N/P in the three soil layers. The SOC, TN, TP, C/N, C/P, and N/P reached the significance level (*P* < 0.05) after HN and HP treatments. Appropriate additions of N and P facilitated absorption and utilization by alfalfa and improved the production potential of alfalfa in alkaline soil.

## Supplementary Information

Supplementary Information 1.

Supplementary Information 2.

## Data Availability

The data that support this study are available from [Plant Stress Research Center, College of Agriculture, Ningxia University], but restrictions apply to the availability of these data, which were used under license for the current study, and so are not publicly available. If the request is reasonable, the test data can be obtained from Professor Xing Xu.
